# Normalization cancer immunotherapy: blocking Siglec-15!

**DOI:** 10.1038/s41392-019-0045-x

**Published:** 2019-04-19

**Authors:** Guangchao Cao, Zhiqiang Xiao, Zhinan Yin

**Affiliations:** 1 The First Affiliated Hospital, Biomedical Translational Research Institute, School of Pharmacy, Jinan University, Guangzhou, China; 2 The Tenth People’s Hospital of Tongji University, Shanghai, China

**Keywords:** Tumour immunology, Immunotherapy

In a recent paper published in *Nature medicine, Wang, J.*, et al. identified Siglec-15 as a new candidate responsible for adaptive immune resistance and a potential target for normalization cancer immunotherapy.^1^

**Fig. 1:**
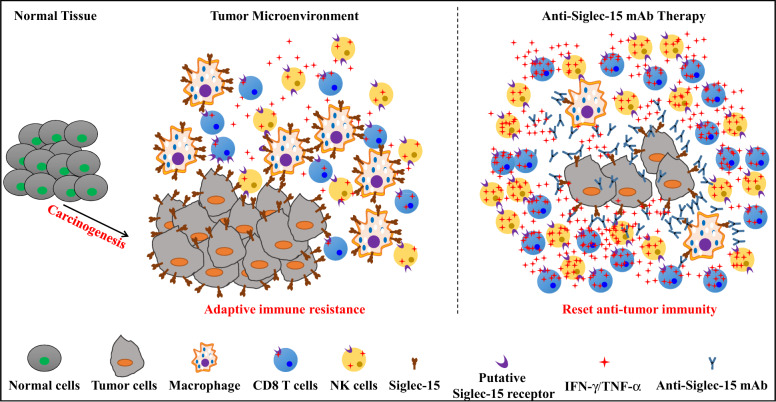
Siglec-15 is a candidate target for normalization cancer immunotherapy. Siglec-15 is minimally expressed on normal tissues at steady-state. However, the expression of Siglec-15 is redundant in a broad spectrum of human cancers and tumor associated myeloid cells. Interestingly, Siglec-15 is mutually exclusive to B7-H1 in human non-small cell lung cancer, which may partially due to its induction by M-GSF and suppression by IFN-γ. Siglec-15 engages with a putative responder protein expressed on effector cells such as CD8 T cells, which induces subsequent suppression of anti-tumor immune responses. Blocking Siglec-15 amplifies anti-tumor immunity in the tumor microenvironment and inhibits tumor growth

Cancer cells can develop various mechanisms to evade tumor-specific and non-specific attacks in the TME of solid tumors.^2^ Thus, systemic activation of immune systems or even increasing tumor-specific T cells in the peripheral does not necessarily lead to tumor regression. In fact, almost all the known strategies adopted by cancer for immune escape are also used for maintaining systemic homeostasis. So, the more non-specific immune responses being activated, the higher risk for adverse events, which may lead to less potential of therapeutic success. The key point is targeting the tumor-induced immune escape mechanisms and these mechanisms should be limited to the tumor microenvironment.^3^ Besides, the immune systems in cancer patients may still provide newly generated effectors, and a “perfect” immunotherapy should prevent the dysfunction of newly generated effectors and selectively restores the immune responses in the TME. Fig. 1.

Blocking PD-L1/PD-1 has been identified as a prototype of normalization cancer immunotherapy. The PD-L1 (B7-H1) protein expression in steady-state normal human tissues is very low, but can be selectively induced in cancer and this induction is limited in the tumor microenvironment. Thus blocking PD-L1/PD-1 avoids systemic overreactions and prevents severe damage to normal tissues, but selectively normalize and reset the immunity in tumor microenvironment.^3^ However, PD-L1/PD-1 is only responsible for a partial of dysfunctional immunity in human solid tumors.^4^ Therefore, identifying new normalizers will increase the spectrum of normalization cancer immunotherapy.

Wang, J., et al. developed a high-throughput genome-scale T cell activity array system (TCAA) to screening cell surface modulators of T cell activities in vitro. Briefly, complementary DNA plasmids coding individual human transmembrane proteins were transfected into an artificial aAPC (293 T cell line that expressed a membrane-associated anti-human CD3 (OKT3) single-chain variable fragment (scFv) and several transmembrane signaling adaptor genes), and co-cultured with Jurkat-NF-κB-GFP or Jurkat-NFAT-GFP cell lines. Thus, transfection of T cell activity stimulators will show enhanced the GFP fluorescence intensity while those inhibitors show decrease intensity in comparison to those transfected with mock. This array system covers 90% of human transmembrane proteins (>6500 genes) and can be carried on 1536-well plates. After a few round of screening and validation, siglec-15 was identified as one of the inhibitory candidates.

Siglec-15 was initially identified as one of the Siglec gene family members with sialic acid-binding immunoglobulin-type lectin structure, and it does bind Sialyl-Tn antigen.^5^ However, it also shows high homology with B7 family members (such as PD-L1/B7-H1), hereby has immune-regulatory potential. Indeed, the authors demonstrate that Siglect-15 is a macrophage-associated T cell immunosuppressive molecule. The mRNA of Siglec-15 is very low in steady-state normal human tissues and most immune cell types, but is ready detectable in macrophages. Similarly, mouse Siglec-15 mRNA is also not detectable in normal tissues, only detectable in BMDMs, but not BMDCs, even upon LPS stimulations. The expression pattern is further validated with specific mAbs against Siglec-15 using flow cytometry, and this protein is only detected on CD11b+ macrophages. In contrast to PD-L1 expression, Siglec-15 expression is inhibited by IFN-γ in vitro. Using both human and mouse Siglec-15 fusion proteins (hS15-hIg and mS15-mIg), the authors further determine a role of Siglec-15 in suppression of T cell activity in vitro using a various assays in both human and mice. More importantly, using OT-1 mice and Siglec-15 KO mice, they elegantly show a suppressive role of Siglec-15 on antigen-specific T cell response in vivo, which is dependent on IL-10. What worth noting was that Siglec-15-KO mice did not develop obvious physical abnormalities, which consistent with rarely expression on normal tissues and suggested minimal adverse effects for Siglec-15 blockade therapy.

Meta-analysis of TCGA database indicated that a broad spectrum of human cancers showed increased Siglec-15 transcription, and this upregulation was validated by in situ mRNA hybridization and Siglec-15-specific Abs staining. Moreover, the expression of Siglec-15 was also abundant on tumor infiltrated macrophages. Interestingly, the expression of Siglec-15 was mutually exclusive to PD-L (B7-H1) in human non-small cell lung cancer (NSCLC), which may partially due to distinct responses to IFN-γ. More importantly, whole-body knockout or macrophages specific knockout of Siglec-15 in mice improved T cells mediated anti-tumor immunity and delayed tumor growth. Finally, anti-Siglec-15 mAbs also blocked the inhibitory effects of Siglec-15 on T cell activities and suppressed the growth of established tumors in mice. Collectively, these results support that Siglec-15 is a potential new candidate target for normalization cancer immunotherapy.

The strict categoria of normalization cancer immunotherapy requires the therapeutic approach to (1) target a mechanism that mediating adaptive immune resistance during carcinogenesis and tumor growth; (2) limit the immune reactions in the TME without inducing systemic adverse effects; and (3) able to reset the anti-tumor immunity in the TME.^3^ Theoretically, blocking Siglec-15 coincides with these requirements. Right now a clinical trial lead by Chen’s group is ongoing to test the effect of an anti-human Siglec-15 mAb (NC318) in solid tumors, which put these exciting findings into practice and brings much expectation.

Although more efforts are needed to clarify the targets of Siglec-15 and the molecular mechanisms of suppression, it is so exciting of discovering this new candidate of tumor adaptive immune resistance. With these unique features of Siglec-15, the anti-Siglec-15 mAb may add to the therapeutic tools for cancer patients, especially to those that are resistant to current anti-PD therapy.
